# Editorial: Computational models of cardiovascular growth and remodeling

**DOI:** 10.3389/fphys.2023.1130420

**Published:** 2023-01-23

**Authors:** Joakim Sundnes, Lik Chuan Lee, Samuel T. Wall, Daniela Valdez-Jasso

**Affiliations:** ^1^ Simula Research Laboratory, Oslo, Norway; ^2^ Department of Mechanical Engineering, Michigan State University, East Lansing, MI, United States; ^3^ Department of Bioengineering, University of California, San Diego, La Jolla, CA, United States

**Keywords:** growth and remodeling, computational physiology, biomechanics, cardiac mechanics, cardiovascular modeling

The cardiovascular system is highly dynamic and responds to mechanical stimuli by changing its anatomy and physiology. This regulation can be compensatory and is important for adapting to the needs of the body. For instance, the heart grows and new collateral blood vessels are formed in response to an increase in myocardial demand with frequent physical exercise. The regulatory process can, however, progress from compensatory to pathological with disease or injury, and it plays a central role in progressive heart failure and chronic heart conditions. Specific mechanical factors, for instance, heart chamber preload (diastolic stretch) and afterload (systolic stress), have been linked to characteristic patterns of cardiac growth and remodeling, and the clinical importance of these and similar findings is widely recognized. The underlying processes, however, are far from fully understood. Important knowledge gaps still exist on a number of different levels. From a viewpoint of fundamental physiology, the mechanisms by which individual cells sense and respond to mechanical stimuli are still poorly understood and remain a target of continuous research. Increased insight into these process can contribute towards a more precise understanding of the key differences between physiological adaptation and pathological maladaptation, which can have direct clinical applications. Other important and largely unresolved questions relate to how the regulatory process of growth and remodeling differs with individual characteristics such as age and sex, and in general how more complex patterns of growth and remodeling can be linked to abnormal mechanical loads.

Computational models of cardiovascular mechanics are used extensively to understand the dynamics of the cardiovascular system, and for quantifying properties that are difficult or impossible to measure experimentally. Successful modeling efforts encompass a wide range of physiological and biomechanical processes, including cardiac electrophysiology, soft tissue mechanics of the heart and vasculature, as well as fluid dynamics of blood flow. More recently, the models have also been extended to describe growth dynamics in the cardiovascular system, and these models stand out as promising research tools for testing hypotheses and gaining insight from sparse experimental data. However, understanding growth and remodeling remains one of the grand challenges of biomechanics, and cardiovascular growth models are still in their infancy. In addition to the general challenges associated with modeling complex physiological processes, models for growth and remodeling face the challenge of highly disparate time scales. Growth and remodeling processes evolve over weeks or several months, while the underlying mechanical and physiological drivers vary at the time scale of a single heart cycle. Effectively bridging these scales, and quantifying the uncertainty associated with the model predictions, is a substantial challenge of mathematical modeling and scientific computing. This Research Topic of Frontiers in Physiology - Computational Physiology and Medicine presents a series of studies that highlight potential clinical applications as well as existing challenges of the cardiovascular growth and remodeling research field.

Predicting potential growth and remodeling is important for a number of clinical interventions to treat cardiovascular disorders. In some cases the goal of the intervention is to induce remodeling or to reverse an ongoing remodeling process, while for other interventions, the goal is to prevent or minimize subsequent remodeling. Valvular intervention is a typical example of the latter, and is addressed in the contribution from Kronborg et al. They present a detailed fluid dynamics model incorporating a decomposition of the flow into components with known and distinct effects on platelet activation and potential blood clot formation. The analysis revealed a close link between mitral valve opening area and risk of clotting. The proposed computational framework can serve as a general tool for studying hemodynamic consequences of cardiovascular interventions as well as growth and remodeling.

Pathological growth and remodeling is triggered or driven by cardiovascular disease or injury, and is often accompanied by inflammation and oedema, which in itself has a severe impact on cardiac function and disease progression. Cardiac oedema results from a complex bio-chemo-mechanical interaction between a pathogen, the immune response, and the balance of mechanical forces that lead to fluid accumulation in the tissue. The paper by de Jesus Lourenço et al. presents a poroelastic model for studying myocardial oedema in acute myocarditis. The model was shown to reproduce known patterns of oedema in myocarditis. Their results also shed light on the distinct roles of key parameters in driving the disease dynamics.

For the last couple of decades, a growing trend in computational cardiovascular research has been to move away from generalized and simplistic models to personalized and patient-specific models. In spite of substantial research effort and a continuous push for models representing *precision medicine*, however, seemingly obvious differences such as those between male and female hearts remain understudied and often neglected. The paper from St. Pierre et al. presents a systematic review of the anatomy, function, and physiological adaptation found in male and female hearts. The study reveals a range of significant differences, and argue for increased research and design of sex-specific diagnostic criteria for earlier and more precise diagnosis of cardiac disease in women.

Hypertension is closely linked to pathological cardiac growth and remodeling. Systemic hypertension is the most common cause of left ventricular hypertrophy, and in recent years we have also witnessed increasing research attention to the impact of pulmonary hypertension on the right ventricle. The contribution by Mendiola et al. extended a finite element model of rat ventricles with a model of the pericardium, and studied its impact on ventricular function in health and disease. The results reveal characteristic differences in how the pericardium restricts the motion of the left and right ventricles, and provide support for previous experimental results on the impact of pericardiectomy on cardiac function. The final contribution in this Research Topic, from Odeigah et al. is also focused on pulmonary hypertension and its effects on right ventricular function. The review provides an overview of the current state-of-the-art in computational models of pulmonary arterial hypertension, including fundamental (patho-)physiological research as well as studies of clinical interventions. The applied computational models include lumped-parameter models of the entire circulatory system, detailed finite element models of ventricular mechanics, and growth and remodeling models that predict the long-term impact of pulmonary hypertension on right ventricular morphology and function.

The handful of papers presented in this Research Topic are clearly not a comprehensive or exhaustive overview of ongoing research in cardiovascular growth and remodeling. However, they provide an interesting display of the breadth of the research field, with computational models ranging from fluid dynamics to multiphysics models of tissue composition. The papers include new research as well as focused and targeted review papers. We hope that these papers will provide new insight and motivate further research into this emerging interdisciplinary research field.

